# Supplementary material for: Modeling glioblastoma heterogeneity as a dynamic network of cell states
Source: Mol Syst Biol. 2021 Sep 16;17(9):e10105. doi: 10.15252/msb.202010105 (PMC8444284; doi:10.15252/msb.202010105)
Supplement: Supplementary file 5 — Source Data for Figure 3 [file MSB-17-e10105-s001.zip › Figure3A_sourcedata/GSEA_3065/nosigdb_state1.GseaPreranked.1622718409535/RICHARDS_NEFTEL_CELL_2019_MES1.html]

Details for gene set RICHARDS\_NEFTEL\_CELL\_2019\_MES1[GSEA]

|  || Dataset | state1 |
| Phenotype | NoPhenotypeAvailable |
| Upregulated in class | na\_pos |
| GeneSet | RICHARDS\_NEFTEL\_CELL\_2019\_MES1 |
| Enrichment Score (ES) | 0.760822 |
| Normalized Enrichment Score (NES) | 2.3507123 |
| Nominal p-value | 0.0 |
| FDR q-value | 0.0 |
| FWER p-Value | 0.0 |
Table: GSEA Results Summary

  

Fig 1: Enrichment plot: RICHARDS\_NEFTEL\_CELL\_2019\_MES1      
 Profile of the Running ES Score & Positions of GeneSet Members on the Rank Ordered List

  

| PROBE | GENE SYMBOL | GENE\_TITLE | RANK IN GENE LIST | RANK METRIC SCORE | RUNNING ES | CORE ENRICHMENT || 1 | LGALS1 |  |  | 4 | 0.828 | 0.0820 | Yes |
| 2 | SPP1 |  |  | 17 | 0.642 | 0.1447 | Yes |
| 3 | TUBA1C |  |  | 24 | 0.619 | 0.2057 | Yes |
| 4 | S100A16 |  |  | 27 | 0.587 | 0.2639 | Yes |
| 5 | ANXA1 |  |  | 30 | 0.564 | 0.3199 | Yes |
| 6 | S100A10 |  |  | 33 | 0.540 | 0.3734 | Yes |
| 7 | ANXA2 |  |  | 48 | 0.482 | 0.4200 | Yes |
| 8 | S100A11 |  |  | 51 | 0.475 | 0.4670 | Yes |
| 9 | PLP2 |  |  | 59 | 0.446 | 0.5107 | Yes |
| 10 | CLIC1 |  |  | 76 | 0.406 | 0.5495 | Yes |
| 11 | PRDX6 |  |  | 79 | 0.398 | 0.5889 | Yes |
| 12 | LGALS3 |  |  | 117 | 0.348 | 0.6198 | Yes |
| 13 | TAGLN2 |  |  | 139 | 0.328 | 0.6504 | Yes |
| 14 | MT2A |  |  | 161 | 0.317 | 0.6798 | Yes |
| 15 | VIM |  |  | 175 | 0.308 | 0.7091 | Yes |
| 16 | GSN |  |  | 213 | 0.290 | 0.7342 | Yes |
| 17 | ACTN1 |  |  | 230 | 0.284 | 0.7608 | Yes |
| 18 | EMP1 |  |  | 473 | 0.211 | 0.7572 | No |
| 19 | IGFBP7 |  |  | 658 | 0.177 | 0.7562 | No |
| 20 | WWTR1 |  |  | 1009 | 0.133 | 0.7338 | No |
| 21 | SERPINE1 |  |  | 1250 | 0.108 | 0.7202 | No |
| 22 | NAMPT |  |  | 1622 | 0.081 | 0.6906 | No |
| 23 | GFPT2 |  |  | 2127 | 0.054 | 0.6447 | No |
| 24 | CD44 |  |  | 2193 | 0.051 | 0.6432 | No |
| 25 | TIMP1 |  |  | 2497 | 0.040 | 0.6163 | No |
| 26 | FN1 |  |  | 2705 | 0.033 | 0.5986 | No |
| 27 | CLIC4 |  |  | 2890 | 0.028 | 0.5826 | No |
| 28 | SOD2 |  |  | 3646 | 0.011 | 0.5070 | No |
| 29 | EFEMP1 |  |  | 4333 | -0.002 | 0.4374 | No |
| 30 | CTSB |  |  | 4873 | -0.011 | 0.3837 | No |
| 31 | MT1X |  |  | 6479 | -0.038 | 0.2244 | No |
| 32 | EMP3 |  |  | 7404 | -0.061 | 0.1365 | No |
| 33 | GJA1 |  |  | 7471 | -0.062 | 0.1360 | No |
| 34 | APOE |  |  | 8322 | -0.095 | 0.0591 | No |
| 35 | TNFRSF1A |  |  | 8416 | -0.100 | 0.0596 | No |
| 36 | NNMT |  |  | 8484 | -0.104 | 0.0631 | No |
| 37 | RCAN1 |  |  | 9149 | -0.158 | 0.0114 | No |
| 38 | NPC2 |  |  | 9235 | -0.170 | 0.0197 | No |
| 39 | C1R |  |  | 9298 | -0.180 | 0.0313 | No |
| 40 | IFITM3 |  |  | 9643 | -0.277 | 0.0240 | No |
Table: GSEA details [plain text format]

  

Fig 2: RICHARDS\_NEFTEL\_CELL\_2019\_MES1: Random ES distribution      
 Gene set null distribution of ES for **RICHARDS\_NEFTEL\_CELL\_2019\_MES1**
